# Intratumor Epigenetic Heterogeneity—A Panel Gene Methylation Study in Thyroid Cancer

**DOI:** 10.3389/fgene.2021.714071

**Published:** 2021-09-03

**Authors:** Chaofan Zhu, Meiying Zhang, Qian Wang, Jin Jen, Baoguo Liu, Mingzhou Guo

**Affiliations:** ^1^Department of Head and Neck Surgery, Peking University Cancer Hospital and Institute, Beijing, China; ^2^Department of Gastroenterology and Hepatology, Chinese PLA General Hospital, Beijing, China; ^3^Genome Analysis Core, Medical Genome Facility, Center for Individualized Medicine, Mayo Clinic, Rochester, MN, United States; ^4^State Key Laboratory of Kidney Diseases, Chinese PLA General Hospital, Beijing, China

**Keywords:** epigenetic heterogeneity, DNA methylation, AP2, CDH1, DACT2, HIN1, RASSF1A

## Abstract

**Background:**

Thyroid cancer (TC) is the most common endocrine malignancy, and the incidence is increasing very fast. Surgical resection and radioactive iodine ablation are major therapeutic methods, however, around 10% of differentiated thyroid cancer and all anaplastic thyroid carcinoma (ATC) are failed. Comprehensive understanding the molecular mechanisms may provide new therapeutic strategies for thyroid cancer. Even though genetic heterogeneity is rigorously studied in various cancers, epigenetic heterogeneity in human cancer remains unclear.

**Methods:**

A total of 405 surgical resected thyroid cancer samples were employed (three spatially isolated specimens were obtained from different regions of the same tumor). Twenty-four genes were selected for methylation screening, and frequently methylated genes in thyroid cancer were used for further validation. Methylation specific PCR (MSP) approach was employed to detect the gene promoter region methylation.

**Results:**

Five genes (*AP2*, *CDH1*, *DACT2*, *HIN1*, and *RASSF1A*) are found frequently methylated (>30%) in thyroid cancer. The five genes panel is used for further epigenetic heterogeneity analysis. *AP2* methylation is associated with gender (*P* < 0.05), *DACT2* methylation is associated with age, gender and tumor size (all *P* < 0.05), HIN1 methylation is associated to tumor size (*P* < 0.05) and extra-thyroidal extension (*P* < 0.01). *RASSF1A* methylation is associated with lymph node metastasis (*P* < 0.01). For heterogeneity analysis, *AP2* methylation heterogeneity *is* associated with tumor size (*P* < 0.01), *CDH1* methylation heterogeneity is associated with lymph node metastasis (*P* < 0.05), *DACT2* methylation heterogeneity is associated with tumor size (*P* < 0.01), *HIN1* methylation heterogeneity is associated with tumor size and extra-thyroidal extension (all *P* < 0.01). The multivariable analysis suggested that the risk of lymph node metastasis is 2.5 times in *CDH1* heterogeneous methylation group (*OR* = 2.512, 95% CI 1.135, 5.557, *P* = 0.023). The risk of extra-thyroidal extension is almost 3 times in *HIN1* heterogeneous methylation group (*OR* = 2.607, 95% CI 1.138, 5.971, *P* = 0.023).

**Conclusion:**

Five of twenty-four genes were found frequently methylated in human thyroid cancer. Based on 5 genes panel analysis, epigenetic heterogeneity is an universal event. Epigenetic heterogeneity is associated with cancer development and progression.

## Introduction

Thyroid cancer (TC) is the most common endocrine malignancy and the incidence is 3.4% of all cancers (Seib and Sosa, 2019). Papillary thyroid cancer (PTC) and follicular thyroid cancer (FTC) are two most common thyroid cancer types, account for 80% and 15% of all thyroid cancer cases. Poorly differentiated thyroid carcinoma (PDTC) and anaplastic thyroid carcinoma (ATC) are account for 5% and 1%, respectively. Surgical resection and radioactive iodine ablation are major therapeutic strategies for thyroid cancer. Treatment failure was observed in about 10% of differentiated thyroid cancer and all ATC patients (Laha et al., 2020; Prete et al., 2020). In the last 30 years, genetic study suggests that the frequency of somatic mutations is relatively low in thyroid cancer (Mazzaferri, 1999). The MAPK and PI3K/Akt pathways were reported to be activated by somatic mutations in thyroid cancer (Mercer and Pritchard, 2003; Ball et al., 2007; Leboeuf et al., 2008; Salerno et al., 2009), and targeted therapies were applied to advanced cancers by tyrosine kinase inhibitors and anti-angiogenic drugs (Yamamoto et al., 2014; Valerio et al., 2017). With the understanding of cancer biology and molecular mechanism, precision medicine gets into the main stage of the era. The key to tailor the management of cancer, including thyroid cancer, is based on the better understanding of the molecular pathways. Landscape of cancer genome study has provided a lot of information of genetic alterations and therapeutic targets for cancer therapy. Mutations in different signaling pathways were found by genomic study in thyroid cancer (Vogelstein et al., 2013; Cha and Koo, 2016). DNA methylation was found frequently in human cancers, including esophageal, colorectal, lung, gastric, and hepatic cancers (Esteller et al., 2002; Brock et al., 2003; House et al., 2003; Guo et al., 2006a,b, 2007, 2008; Licchesi et al., 2008; Wang et al., 2009, 2017; Jia et al., 2012, 2013; Hu et al., 2015; Li et al., 2015; Zheng et al., 2017). However, epigenetics was not extensively studied in thyroid cancer. Pan-cancer landscape of aberrant DNA methylation across 24 cancers demonstrated that the methylation frequency was lowest in PTC (Saghafinia et al., 2018). Aberrant methylation of *SOX17* and *DACT2*, the key components of Wnt signaling, were found frequently in thyroid cancer (Li et al., 2012; Zhao et al., 2014). Methylation of *GPX3* was found associated with thyroid cancer metastasis (Zhao et al., 2015). Phenotypic and functional heterogeneity are hallmarks of human cancers (Visvader, 2011). Subpopulations of cancer cells with distinct phenotypic and molecular features within a tumor are called intratumor heterogeneity (ITH) (Bedard et al., 2013). For cancer genetic study, researchers mainly focused on mutational activation of oncogenes or inactivation of tumor-suppressor genes (TSGs). The theory of Darwinian-like clonal evolution of a single tumor was employed to explain the phenomenon of ITH (Nowell, 1976). However, the dominance of gene-centric views is challenged by cancer stem cell hypothesis, and phenotypic variability becomes the major topic of cancer research (Marusyk et al., 2012). The concept of epigenetic silencing being involved in Knudson’s two-hit theory was accepted and the causal relevance of epigenetic changes in cancer is being recognized (Nagasaka et al., 2010). The occurrence of abnormal epigenetic change is more frequently than driver mutations in human cancer. In the process of cancer initiation and development, disruption of the “epigenetic machinery” plays an important role. The disruption of “epigenetic machinery” may contribute to tumor phenotype heterogeneity (Guo et al., 2019).

ITH plays an important role in chemotherapeutic resistance (Guo et al., 2019). The best regimen for cancer therapy is to target all different subpopulations of cancer cells at the same time, to avoid chemo-resistance and reduce relapse (Pribluda et al., 2015). Therapeutic failures are often attributed to adaptive responses of cancer stem cells. Environmental and therapeutic pressures may drive transcriptional plasticity through the response of epigenetic regulators to cause durable disease remission in patients for many cancer therapeutic drugs (Dawson, 2017). Epigenetic heterogeneity is more dynamic compare to genetic heterogeneity. Cancer epigenetic heterogeneity is in its infancy and there are very limited studies involved in epigenetic heterogeneity (Dawson, 2017; Guo et al., 2019).

In this study, we evaluated epigenetic heterogeneity by examining promoter region methylation in a panel of genes in primary thyroid cancer. These genes are frequently methylated in thyroid and other cancers, and they are involved in different cancer-related signaling.

## Materials and Methods

### Patients and Specimens

A total of 405 samples from 135 cases of thyroid cancer (46 cases were served as discovery group for methylation screening and all cases were served as validation group) were obtained in Beijing Cancer Hospital from 2018 to 2020 (Supplementary Table 1). For heterogeneity analysis, a total of 405 surgical resected thyroid cancer samples were employed (three spatially isolated specimens were obtained from different regions of the same tumor). The median age was 42 years old (range 24–79 years old), including 104 cases of female and 31 cases of male patients. Cancer samples were classified according to the TNM staging system (AJCC2018), including tumor stage I (*n* = 130), stage II (*n* = 4), and stage III (*n* = 1). All of the tissue samples were immediately snap frozen in liquid nitrogen and preserved at −80°C before analysis. All samples were collected following the guidelines approved by the Institutional Review Board of the Beijing Cancer Hospital.

### DNA Extraction, Bisulfite Modification, Methylation Specific PCR, and Screening for Representative Genes

Genomic DNA was extracted from frozen tissues, digested with protease K and then extracted using the standard phenol/chloroform procedure (Cao et al., 2013). Methylation-specific PCR (MSP) primers were designed according to genomic sequences around transcriptional start sites (TSS) and synthesized to detect unmethylated (U) and methylated (M) alleles. Bisulfite treatment was performed as previously described (Herman et al., 1996). MSP primers were listed in Supplementary Table 2. MSP amplification conditions were as follows: 95°C 5 min, 1 cycle; 95°C 30 s, 60°C 30 s, 72°C 40 s, 35 cycles; 72°C 5 min, 1 cycle.

To screen representative genes for heterogeneity analysis, twenty-four genes were selected as discovery group, including *MGMT, TIMP3, DAPK, MLH1, TMEM176A, DIRAS1, SFRP1, SFRP2, HIN1, AP2, ER, DACT2, CDH1, RASSF1A, SOX17, GATA4, BCL6B, GPX3, CRBP-1, p16, RUNX3, RAR*β, *CDX2*, and *WIF1*. All these genes were found frequently methylated in esophageal, colorectal, lung, gastric and hepatic cancers by our previous studies and others, except for *AP2* (an important transcription factor). These genes were well characterized and reported to be involved in different signaling pathways. Among which, *AP2, CDH1, DACT2, HIN1*, and *RASSF1A* genes were found frequently methylated (>30%) and selected for heterogeneity analysis. The workflow is shown in Supplementary Figure 1.

### Statistical Analysis

The Chi-square or Fisher exact-test was used to analyze the association of gene methylation status and clinical factors. Logistic regression analysis was used to analyze the association of methylation heterogeneity or clinical factors with lymph node Metastasis and Extrathyroidal extension. *P* < 0.05^∗^, *P* < 0.01^∗∗^, or *P* < 0.001^∗∗∗^ was regarded as statistically significant. Data were analyzed by SPSS 22.0 software.

## Results

### Selection of Frequently Methylated Genes in Human Thyroid Cancer

**FIGURE 1:**
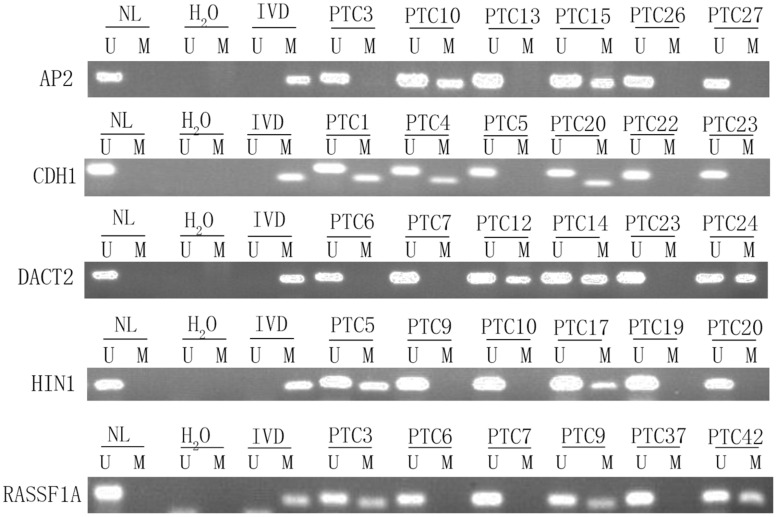
Representative methylation results of *AP2*, *HIN1*, *DACT2, RASSF1A*, and *CDH1* in thyroid cancer (discovery group). IVD, *in vitro*-methylated DNA (methylation control); NL, normal lymphocyte DNA (unmethylation control); H_2_O, double distilled water; U, unmethylation; M, methylation; PTC, papillary thyroid cancer.

To explore epigenetic heterogeneity in thyroid cancer, 24 genes, which were found frequently methylated in other cancers, were selected as discovery group to detect 46 cases of thyroid cancer. As shown in Table 1 and Figure 1, the methylation rate is 0% (0/46)–56.52% (26/46). Five genes (*AP2*, *CDH1*, *DACT2*, *HIN1*, and *RASSF1A*) are methylated more than 30% in thyroid cancer. These five genes panel is selected as validation group for methylation heterogeneity analysis.

**TABLE 1:** Methylation status of 24 genes in discovery group.

Gene	Thyroid cancer (*n* = 46)
*MGMT*	0%(0/46)
*TIMP3*	0%(0/46)
*DAPK*	2.17%(1/46)
*MLH1*	2.17%(1/46)
*TMEM176A*	23.91%(11/46)
*DIRAS1*	28.26%(13/46)
*sFRP1*	28.26%(13/46)
*sFRP2*	10.87%(5/46)
*HIN1*	32.61%(15/46)
*AP2*	34.78%(16/46)
*ER*	4.35%(2/46)
*DACT2*	45.65%(21/46)
*CDH1*	47.82%(22/46)
*RASSF1A*	56.52%(26/46)
*SOX17*	8.70%(4/46)
*GATA4*	8.70%(4/46)
*BCL6B*	28.26%(13/46)
*GPX3*	17.39%(8/46)
*CRBP-1*	0%(0/46)
*p16*	2.17%(1/46)
*RUNX3*	2.17%(1/46)
*RAR*β	4.35%(2/46)
*CDX2*	6.52%(3/46)
*WIF1*	10.87%(5/46)

### Intratumor Epigenetic Heterogeneity in Thyroid Cancer

To evaluate the intratumor epigenetic heterogeneity in thyroid cancer, three different tumor samples are obtain from isolated locations in the same patient. Totally 405 cancer samples are gained from 135 patients. The methylation status of *AP2*, *CDH1*, *DACT2*, *HIN1*, and *RASSF1A* genes is examined by MSP.

The association of promoter region methylation and clinical factors is analyzed by Chi-square tests, including gender, age, tumor size, tumor location, TNM stage, lymph node metastasis, and extra-thyroidal extension. As shown in Table 2, *AP2* methylation is associated with gender (*P* < 0.05), while no association were found between *AP2* methylation and age, tumor size, tumor location, TNM stage, lymph node metastasis, and extra-thyroidal extension (all *P* > 0.05). No association are found between *CDH1* methylation and gender, age, tumor size, tumor location, TNM stage, lymph node metastasis, and extra-thyroidal extension (all *P* > 0.05). *DACT2* methylation is associated with age, gender and tumor size (all *P* < 0.05), while no association is found between *DACT2* methylation and tumor location, TNM stage, lymph node metastasis, and extra-thyroidal extension (all *P* > 0.05). *HIN1* methylation is associated with tumor size (*P* < 0.05) and extra-thyroidal extension (*P* < 0.01), while no association is found between *HIN1* methylation and age, tumor location, TNM stage, and lymph node metastasis (all *P* > 0.05). *RASSF1A* methylation is associated with lymph node metastasis (*P* < 0.01), while no association is found between *RASSF1A* methylation and age, tumor size, tumor location, TNM stage, and extra-thyroidal extension (all *P* > 0.05).

**TABLE 2:** The association of gene methylation and clinical factors in validation group.

		*AP2*			CDH1			DACT2			HIN1			*RASSF1A*		
						
	No. *n* = 135	*M n* = *94*	U n = 41	*p*	M n = 75	U n = 60	*p*	M n = 96	U n = 39	*p*	M n = 100	U n = 35	*p*	M n = 114	U n = 21	*p*
**Age (year)**																
<55	114	77 (67.5%)	37 (32.5%)	0.219	63 (55.3%)	51 (44.7%)	0.873	77 (67.5%)	37 (32.5%)	0.033*	83 (72.8%)	31 (27.2%)	0.434	95 (83.3%)	19 (16.7%)	0.615
≥55	21	17 (81.0%)	4 (19.0%)		12 (57.1%)	9 (42.9%)		19 (90.5%)	2 (9.5%)		17 (81.0%)	4 (19.0%)		19 (90.5%)	2 (9.5%)	
**Gender**																
Male	31	17 (54.8%)	14 (45.2%)	0.041*	16 (51.6%)	15 (48.4%)	0.615	17 (54.8%)	14 (45.2%)	0.023*	22 (71.0%)	9 (29.0%)	0.653	29 (93.5%)	2 (6.5%)	0.196
Female	104	77 (74.0%)	27 (26.0%)		59 (56.7%)	45 (43.3%)		79 (76.0%)	25 (24.0%)		78 (75.0%)	26 (25.0%)		85 (81.7%)	19 (18.3%)	
**Tumor size (cm)**																
≤1 cm	103	75 (72.8%)	28 (27.2%)	0.149	57 (55.3%)	46 (44.7%)	0.928	78 (75.7%)	25 (24.3%)	0.034*	81 (78.6%)	22 (21.4%)	0.030*	86 (83.5%)	17 (16.5%)	0.585
>1 cm	32	19 (59.4%)	13 (40.6%)		18 (56.25%)	14 (43.75%)		18 (56.25%)	14 (43.75%)		19 (59.4%)	13 (40.6%)		28 (87.5%)	4 (12.5%)	
**Tumor location**																
Left lobe	68	46 (67.6%)	22 (32.4%)	0.614	38 (55.9%)	30 (44.1%)	0.939	49 (72.1%)	19 (27.9%)	0.807	53 (77.9%)	15 (22.1%)	0.302	56 (82.4%)	12 (17.6%)	0.499
Right lobe	67	48 (71.6%)	19 (28.4%)		37 (55.2%)	30 (44.8%)		47 (70.1%)	20 (29.9%)		47 (70.1%)	20 (29.9%)		58 (86.6%)	9 (13.4%)	
**TNM stage**																
I	130	90 (69.2%)	40 (30.8%)	0.985	72 (55.4%)	58 (44.6%)	1.000	91 (70.0%)	39 (30.0%)	0.342	97 (74.6%)	33 (25.4%)	0.832	110 (84.6%)	20 (15.4%)	0.577
II + III	5	4 (80.0%)	1 (20.0%)		3 (60.0%)	2 (40.0%)		5 (100.0%)	0 (0.0%)		3 (60.0%)	2 (40.0%)		4 (80.0%)	1 (20.0%)	
**LNM**																
N0	76	56 (73.7%)	20 (26.3%)	0.245	45 (59.2%)	31 (40.8%)	0.332	57 (75.0%)	19 (25.0%)	0.258	57 (75.0%)	19 (25.0%)	0.781	70 (92.1%)	6 (7.9%)	0.005**
N1	59	38 (64.4%)	21 (35.6%)		30 (50.8%)	29 (49.2%)		39 (66.1%)	20 (33.9%)		43 (72.9%)	16 (27.1%)		44 (74.6%)	15 (25.4%)	
**Extrathyroidal extension**																
*Negative*	91	64 (70.3%)	27 (29.7%)	0.799	51 (56.0%)	40 (44.0%)	0.870	67 (73.6%)	24 (26.4%)	0.354	74 (81.3%)	17 (18.7%)	0.006**	78 (85.7%)	13 (14.3%)	0.558
*Positive*	44	30 (68.2%)	14 (31.8%)		24 (54.5%)	20 (45.5%)		29 (65.9%)	15 (34.1%)		26 (59.1%)	18 (40.9%)		36 (81.8%)	8 (18.2%)	

M, methylation; U, unmethylation; LNM, lymph node metastasis; Chi-square test and Fisher exact test, *p < 0.05, **p < 0.01.

**FIGURE 2:**
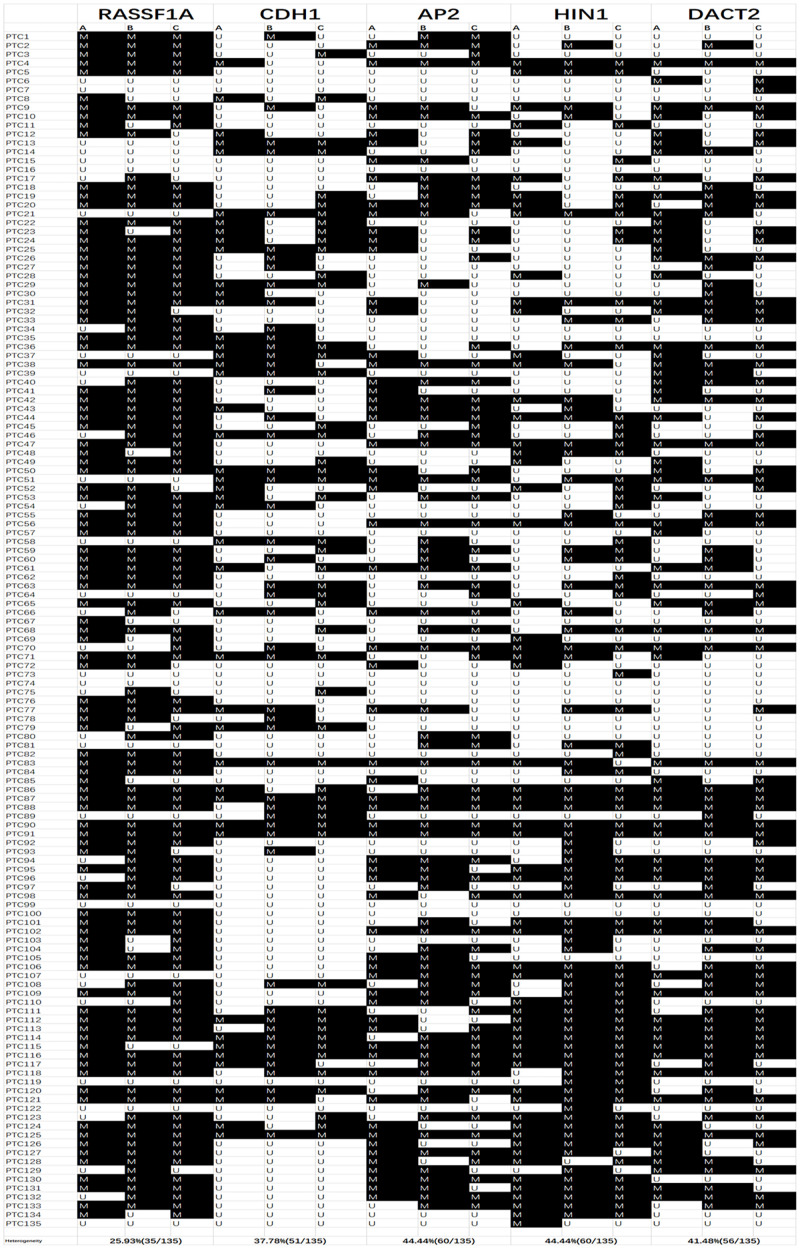
Methylation pattern of 3 different samples for each patient. M, methylation; U, unmethylation; Colum (A–C) are 3 different samples for each case; PTC, papillary thyroid cancer.

**FIGURE 3:**
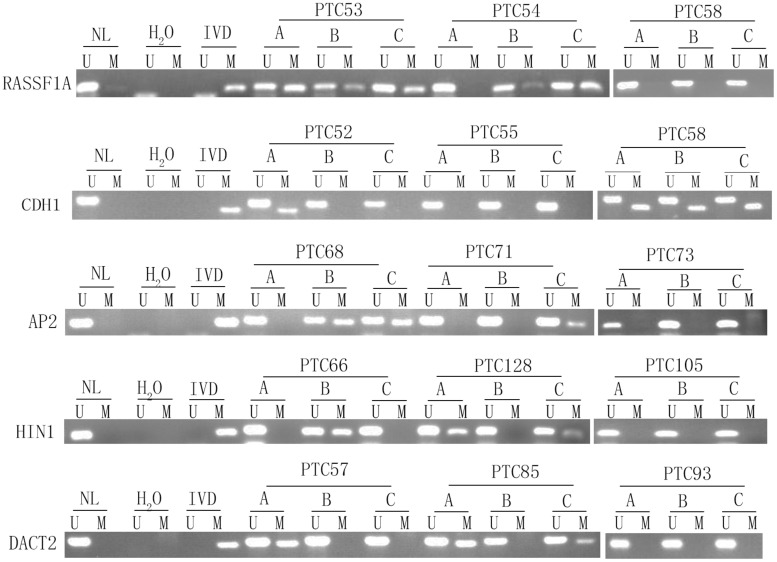
Representative methylation heterogeneity results for each of five genes. IVD, *in vitro*-methylated DNA (methylation control); NL, normal lymphocyte DNA (unmethylation control); H_2_O, double distilled water; U, unmethylation; M, methylation; PTC, papillary thyroid cancer.

Unmethylation or methylation in all three samples from the same individual is regarded as homogeneity, while unmethylation or methylation in one or two samples from the same individual is regarded as methylation heterogeneity. As shown in Figures 2, 3, methylation heterogeneity is found in 25.93% (35/135), 37.78% (51/135), 44.44% (60/135), 44.44% (60/135), and 41.48% (56/135) of cases for *RASSF1A*, *CDH1*, *AP2*, *HIN1*, and *DACT2* genes. The results suggest that methylation heterogeneity is a common event in human thyroid cancer.

The association of methylation heterogeneity and clinical factors is further analyzed. As shown in Table 3, *AP2* methylation heterogeneity is associated with tumor size (*P* < 0.01), no association are found between *AP2* methylation heterogeneity and gender, age, tumor location, TNM stage, lymph node metastasis and extra-thyroidal extension (all *P* > 0.05). *CDH1* methylation heterogeneity is associated with lymph node metastasis (*P* < 0.05), no association are found between *CDH1* methylation heterogeneity and gender, age, tumor size, tumor location, TNM stage and extra-thyroidal extension (all *P* > 0.05). *DACT2* methylation heterogeneity is associated with tumor size (*P* < 0.01), no association are found between *DACT2* methylation heterogeneity and gender, age, tumor location, TNM stage, lymph node metastasis and extra-thyroidal extension (all *P* > 0.05). *HIN1* methylation heterogeneity is associated with tumor size and extra-thyroidal extension (all *P* < 0.01), no association are found between *HIN1* methylation heterogeneity and gender, age, tumor location, TNM stage and lymph node metastasis (all *P* > 0.05). No association are found between *RASSF1A* methylation heterogeneity and gender, age, tumor size, tumor location, TNM stage, lymph node metastasis and extra-thyroidal extension (all *P* > 0.05). Above results demonstrate that methylation heterogeneity of three genes (*AP2*, *DACT2*, and *HIN1*) is associated with tumor size.

**TABLE 3:** The association of methylation heterogeneity and clinical factors.

		*AP2*			*CDH1*			*DACT2*			*HIN1*			*RASSF1A*		
						
	*No. n* = *135*	*HE n* = *60*	*HO n* = *75*	*p*	*HE n* = *51*	*HO n* = *84*	*p*	*HE n* = *56*	*HO n* = *79*	*p*	*HE n* = *60*	*HO n* = *75*	*p*	*HE n* = *35*	*HO n* = *100*	*p*
**Age (year)**																
<55	114	50	64	0.750	43	71	0.974	44	70	0.113	51	63	0.873	31	83	0.434
≥55	21	10	11		8	13		12	9		9	12		4	17	
**Gender**																
Male	31	13	18	0.749	14	17	0.334	13	18	0.953	13	18	0.749	10	21	0.359
Female	104	47	57		37	67		43	61		47	57		25	79	
**Tumor size (cm)**																
≤1 cm	103	53	50	0.003**	40	63	0.649	50	53	0.003**	53	50	0.003**	28	75	0.549
>1 cm	32	7	25		11	21		6	26		7	25		7	25	
**Tumor location**																
Left lobe	68	28	40	0.441	26	42	0.912	29	39	0.782	29	39	0.672	15	53	0.302
Right lobe	67	32	35		25	42		27	40		31	36		20	47	
**TNM stage**																
I	130	58	72	1.000	50	80	0.651	54	76	1.000	59	71	0.508	33	97	0.604
II + III	5	2	3		1	4		2	3		1	4		2	3	
**Lymph node metastasis**																
N0	76	32	44	0.535	35	41	0.024*	35	41	0.221	31	45	0.332	20	56	0.907
N1	59	28	31		16	43		21	38		29	30		15	44	
**Extrathyroidal extension**																
Negative	91	41	50	0.837	35	56	0.814	41	50	0.226	48	43	0.005**	25	66	0.555
Positive	44	19	25		16	28		15	29		12	32		10	34	

HE, heterogeneity; HO, homogeneity;Chi square test, *p < 0.05, **p < 0.01.

The association of DNA methylation heterogeneity and lymph node metastasis or extra-thyroidal extension is further analyzed by logistic regression model. Univariate logistic analysis indicated that *CDH1* methylation heterogeneity, age and tumor size are associated with lymph node metastasis, independently (*P* = 0.026, *P* = 0.000, *P* = 0.040, Table 4). *HIN1* heterogeneous methylation, tumor size and tumor location are associated with extra-thyroidal extension, independently (*P* = 0.006, *P* = 0.004, *P* = 0.025, Table 5). Interesting, the multivariable analysis suggested that the risk of lymph node metastasis is 2.5 times in *CDH1* heterogeneous methylation group compare to *CDH1* homogeneous methylation group (*OR* = 2.512, 95% CI 1.135, 5.557, *P* = 0.023, Table 4). The risk of extra-thyroidal extension is almost 3 times in *HIN1* heterogeneous methylation group than in *HIN1* homogeneous methylation group (*OR* = 2.607, 95% CI 1.138, 5.971, *P* = 0.023, Table 5). The results indicate that with the growing of tumor size, the phenotype of tumor cell becomes diversity. Epigenetic herterogeneity may reflect different phenotypes of population of tumor cells and increased the risk of tumor metastasis significantly. Epigenetic herterogeneity may provide more information for tumor therapeutic strategies.

**TABLE 4:** Logistic regression model for lymph node metastasis.

Variable	Univariate analysis		Multivariate analysis	
		
	OR (95% CI)	*p*	OR (95% CI)	*p*
AP2 (HE vs. HO)	0.805(0.406,1.596)	0.535		
CDH1 (HE vs. HO)	2.294(1.106,4.761)	0.026*	2.512(1.135,5.557)	0.023*
DACT2 (HE vs. HO)	1.545(0.768,3.105)	0.222		
HIN1 (HE vs. HO)	0.713(0.359,1.414)	0.333		
RASSF1A (HE vs. HO)	1.048(0.482,2.279)	0.907		
Age (24–79 years)	0.933(0.898,0.969)	< 0.001***	0.927(0.891,0.965)	< 0.001***
Gender (female vs. male)	1.080(0.482,2.419)	0.852		
Tumor size (0.2–3 cm)	2.190(1.037,4.623)	0.040*	2.429(1.086,5.436)	0.031*
Tumor location (Right lobe vs. Left lobe)	1.167(0.591,2.305)	0.657		
TNM Stage (II + III vs. I)	0.183(0.020,1.686)	0.134		
Extrathyroidal extension (positive vs. negative)	1.273(0.618,2.626)	0.513		

HE, heterogeneity methylation; HO, homogeneity methylation; *p < 0.05, ***p < 0.001.

**TABLE 5:** Logistic regression analysis for extrathyroidal extension.

Variable	Univariate analysis		Multivariate analysis	
		
	OR (95% CI)	*p*	OR (95% CI)	*p*
AP2 (HE vs. HO)	1.079(0.522,2.229)	0.837		
CDH1 (HE vs. HO)	1.094(0.519,2.305)	0.814		
DACT2 (HE vs. HO)	1.585(0.751,3.349)	0.227		
HIN1 (HE vs. HO)	2.977(1.364,6.498)	0.006**	2.607(1.138,5.971)	0.023*
RASSF1A (HE vs. HO)	1.288(0.555,2.989)	0.556		
Age (24–79 years)	1.012(0.979,1.047)	0.477		
Gender (female vs. male)	2.031(0.890,4.634)	0.092		
Tumor size (0.2–3 cm)	3.249(1.465,7.206)	0.004**	2.859(1.224,6.679)	0.015*
Tumor location (Right lobe vs. Left lobe)	0.429(0.204,0.900)	0.025*	0.373(0.168,0.828)	0.015*
TNM stage (II + III vs. I)	0.716(0.115,4.448)	0.720		
Lymph node metastasis (positive vs. negative)	1.273(0.618,2.626)	0.513		

HE, heterogeneity methylation; HO, homogeneity methylation; *p < 0.05, **p < 0.01.

## Discussion

Genomic heterogeneity has been well studied in various cancers (Burrell et al., 2013; Hiley et al., 2016; Maura et al., 2021; Nicholson and Fine, 2021; Wand and Lambert, 2021). However, there are a very few reports about epigenetic heterogeneity in human cancers. Our previous study found that “a field defect of epigenetic changes” was presented in bronchial margins of surgical resected lung cancer samples (Guo et al., 2004). By detecting the methylation status of five genes (*RASSF1A*, *p16*, *DAPK*, *MGMT*, and *Rb*) in 34 tumors (including 15 melanoma primaries, 19 metastases), heterogeneous methylation was found in 70% of the cases (Rastetter et al., 2007). Among 9 MSI-positive primary endometrial cancers lack of MLH1 expression, which was evaluated by immunohistochemistry, Varley et al. (2009) found that 8 cases were methylated in the promoter region. In the 8 cases of methylated patients, four cases were heterogeneously methylated and four tumors were homogeneously methylated. In the discovery group, we select 24 genes, which were well characterized and frequently methylated in various cancers. Five genes are found frequently methylated (> 30%) in 46 cases of human thyroid cancer. In the validation group, total of 405 samples are included (135 cases of patients and 3 samples from different location of each tumor). Among these five genes, *DACT2* methylation is associated with gender, age, and tumor size (all *P* < 0.05), *HIN1* methylation is associated with tumor size (*P* < 0.05) and extra-thyroidal extension (*P* < 0.01), *RASSF1A* methylation is associated with lymph node metastasis (*P* < 0.01). The results suggest that methylation of DACT2, HIN1, and RASSF1A increases the malignance of thyroid cancer. Further analysis find that methylation heterogeneity of *AP2*, *DACT2*, and *HIN1* are associated with tumor size. The results suggest that epigenetic heterogeneity is increasing with tumor growth. It indicates that the phenotype of cancer cells is varied in different tumor stages. Thus, the therapeutic strategies need according to the phenotypes of cancer cells, which are determined by epigenetic changes. The multivariable analysis suggested that *CDH1* methylation heterogeneity is associated with lymph node metastasis and *HIN1* methylation heterogeneity is associated with extra-thyroidal extension. The results suggest that *CDH1* and *HIN1* methylation heterogeneity may increase tumor metastasis. Methylation heterogeneity of these two genes may serve as prognostic marker for thyroid cancer.

The conventional clinical therapeutics is the “one-size-fits-all-approach.” However, the ultimate aim of precision medicine is to enable clinicians to accurately and efficiently identify the most effective preventative or therapeutic intervention for a specific patient. Epigenetic switches play important roles in carcinogenesis and tumor progression, and epigenetic switches are reversible (Guo et al., 2019). In this study, we focus mainly on the epigenetic heterogeneity of transcriptional regulators, which were found involved in different cancer-related signaling pathways. Epigenetic heterogeneity is found in four (*CDH1*, *AP2*, *HIN1*, and *DACT2*) of the five detected genes in thyroid cancer. The results suggest that epigenetic heterogeneity is a universal mechanism of cancer development. It is notable that epigenetic heterogeneity is associated with tumor size or tumor metastasis. For the first time, we find that epigenetic heterogeneity is related to cancer development. Based on “BRCAness” principle, our recent study found that methylation of *NRN1* was a novel synthetic lethal marker for PI3K-Akt-mTOR and ATR inhibitors in human esophageal cancer (McLornan et al., 2014; Lord and Ashworth, 2016, 2017; Du et al., 2021). It is reasonable to tailor the regimen for each individual of cancer patients by epigenetic changes or epigenetic heterogeneity.

In conclusion, 5 of 24 genes were found frequently methylated in human thyroid cancer. Based on the 5 genes panel analysis, epigenetic heterogeneity is an universal event. Epigenetic heterogeneity is associated with cancer development and progression.

## Data Availability Statement

The original contributions presented in the study are included in the article/Supplementary Material, further inquiries can be directed to the corresponding author/s.

## Ethics Statement

All samples were collected following the guidelines approved by the Institutional Review Board of the Beijing Cancer Hospital. The patients/participants provided their written informed consent to participate in this study.

## Author Contributions

CZ, MZ, and QW designed the project and performed the experiments. MZ and MG wrote the manuscript. MG has made significant contributions to the concept of this study. BL and JJ take part in data interpretation. All authors read and approved the final manuscript.

## Conflict of Interest

The authors declare that the research was conducted in the absence of any commercial or financial relationships that could be construed as a potential conflict of interest.

## Publisher’s Note

All claims expressed in this article are solely those of the authors and do not necessarily represent those of their affiliated organizations, or those of the publisher, the editors and the reviewers. Any product that may be evaluated in this article, or claim that may be made by its manufacturer, is not guaranteed or endorsed by the publisher.

## Supplementary Material

Supplementary Figure 1 The workflow of epigenetic heterogeneity analysis.

Supplementary Table 1 Clinical factors of patients.

Supplementary Table 2 Primers used for methylation analysis.
