# Supplementary figures and images for: Intratumor Epigenetic Heterogeneity—A Panel Gene Methylation Study in Thyroid Cancer
Source: Front Genet. 2021 Sep 3;12:714071. doi: 10.3389/fgene.2021.714071 (PMC8446600; doi:10.3389/fgene.2021.714071)

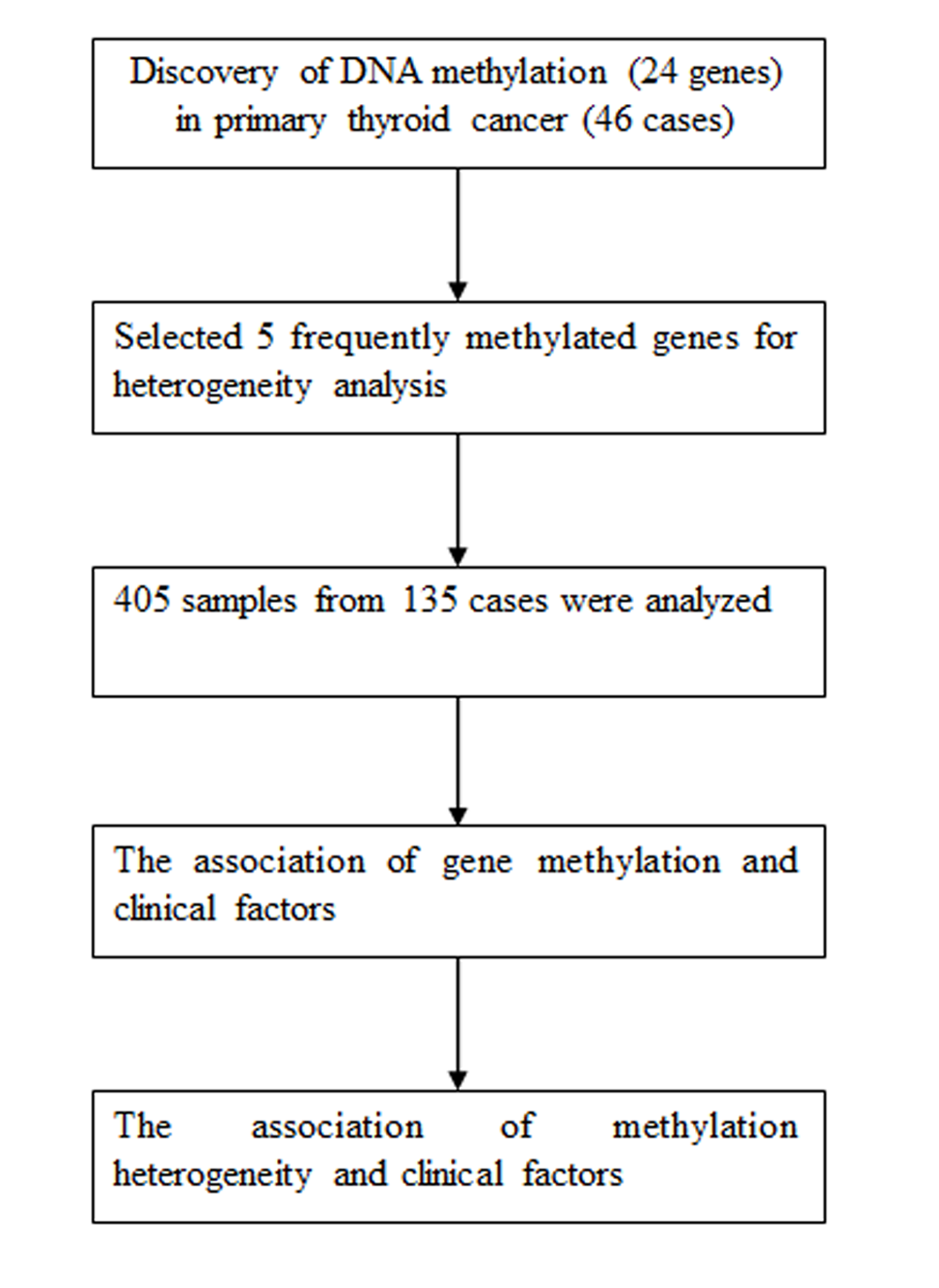

Supplement: Supplementary Figure 1 — The workflow of epigenetic heterogeneity analysis. [file Image_1.TIF]
