# Supplementary material for: Intratumor Epigenetic Heterogeneity—A Panel Gene Methylation Study in Thyroid Cancer
Source: Front Genet. 2021 Sep 3;12:714071. doi: 10.3389/fgene.2021.714071 (PMC8446600; doi:10.3389/fgene.2021.714071)
Supplement: Supplementary Table 1 — Clinical factors of patients. [file Table_1.docx]

Supplementary Table 1: Clinical factors of patients

| **Patient** | **Age** | **Gender** | **Tumor size(cm)** | **Tumor location** | **TNM stage** | **Lymph node Metastasis** | **Extrathyroidal extension** |
| --- | --- | --- | --- | --- | --- | --- | --- |
| 1 | 32 | Female | 0.5 | Right lobe | I | Negative | Negative |
| 2 | 60 | Female | 0.5 | Right lobe | I | Negative | Negative |
| 3 | 53 | Female | 1 | Right lobe | I | Negative | Negative |
| 4 | 44 | Female | 1 | Right lobe | I | Negative | Negative |
| 5 | 35 | Male | 0.8 | Left lobe | I | Positive | Negative |
| 6 | 36 | Female | 0.5 | Right lobe | I | Negative | Negative |
| 7 | 30 | Female | 1 | Right lobe | I | Positive | Negative |
| 8 | 50 | Female | 2 | Right lobe | I | Positive | Positive |
| 9 | 29 | Female | 0.6 | Left lobe | I | Positive | Negative |
| 10 | 55 | Female | 0.8 | Right lobe | I | Negative | Negative |
| 11 | 33 | Female | 0.8 | Right lobe | I | Positive | Negative |
| 12 | 57 | Female | 0.5 | Left lobe | I | Negative | Negative |
| 13 | 53 | Female | 0.3 | Right lobe | I | Negative | Negative |
| 14 | 56 | Female | 0.6 | Left lobe | II | Positive | Negative |
| 15 | 30 | Female | 0.8 | Right lobe | I | Positive | Negative |
| 16 | 41 | Female | 1 | Right lobe | I | Positive | Positive |
| 17 | 45 | Male | 0.8 | Right lobe | I | Negative | Negative |
| 18 | 35 | Female | 0.6 | Left lobe | I | Positive | Negative |
| 19 | 42 | Female | 0.7 | Left lobe | I | Positive | Negative |
| 20 | 53 | Female | 0.8 | Left lobe | I | Negative | Negative |
| 21 | 29 | Female | 0.3 | Left lobe | I | Positive | Negative |
| 22 | 55 | Female | 1.2 | Right lobe | II | Negative | Positive |
| 23 | 40 | Female | 0.2 | Right lobe | I | Positive | Negative |
| 24 | 28 | Male | 0.7 | Left lobe | I | Positive | Negative |
| 25 | 42 | Female | 1.5 | Right lobe | I | Negative | Negative |
| 26 | 33 | Female | 0.7 | Right lobe | I | Negative | Positive |
| 27 | 42 | Male | 1.2 | Left lobe | I | Positive | Positive |
| 28 | 46 | Female | 0.2 | Left lobe | I | Negative | Negative |
| 29 | 27 | Male | 0.3 | Right lobe | I | Negative | Positive |
| 30 | 59 | Female | 0.5 | Left lobe | I | Negative | Positive |
| 31 | 59 | Female | 1 | Right lobe | I | Negative | Negative |
| 32 | 63 | Female | 0.7 | Right lobe | III | Positive | Positive |
| 33 | 45 | Female | 0.7 | Left lobe | I | Positive | Negative |
| 34 | 40 | Male | 1.7 | Left lobe | I | Negative | Positive |
| 35 | 35 | Male | 1.2 | Right lobe | I | Negative | Negative |
| 36 | 48 | Female | 1.4 | Left lobe | I | Positive | Negative |
| 37 | 51 | Female | 0.7 | Right lobe | I | Negative | Positive |
| 38 | 40 | Female | 1.8 | Left lobe | I | Positive | Negative |
| 39 | 36 | Female | 0.3 | Left lobe | I | Positive | Negative |
| 40 | 45 | Female | 0.3 | Right lobe | I | Negative | Positive |
| 41 | 33 | Male | 0.6 | Right lobe | I | Positive | Positive |
| 42 | 43 | Female | 0.2 | Right lobe | I | Positive | Negative |
| 43 | 52 | Female | 1.5 | Right lobe | I | Negative | Positive |
| 44 | 46 | Female | 0.3 | Right lobe | I | Negative | Negative |
| 45 | 28 | Female | 1 | Left lobe | I | Positive | Negative |
| 46 | 31 | Female | 1 | Right lobe | I | Positive | Positive |
| 47 | 70 | Male | 1.5 | Left lobe | I | Negative | Negative |
| 48 | 33 | Female | 0.6 | Left lobe | I | Positive | Negative |
| 49 | 40 | Male | 1 | Left lobe | I | Positive | Negative |
| 50 | 30 | Female | 0.5 | Left lobe | I | Positive | Negative |
| 51 | 54 | Female | 0.8 | Right lobe | I | Positive | Positive |
| 52 | 56 | Female | 0.5 | Right lobe | I | Negative | Negative |
| 53 | 46 | Male | 0.5 | Right lobe | I | Negative | Negative |
| 54 | 32 | Male | 0.6 | Right lobe | I | Positive | Negative |
| 55 | 55 | Female | 0.7 | Left lobe | I | Negative | Negative |
| 56 | 53 | Female | 1.3 | Right lobe | I | Negative | Positive |
| 57 | 50 | Female | 1 | Right lobe | I | Positive | Positive |
| 58 | 36 | Female | 0.7 | Left lobe | I | Positive | Positive |
| 59 | 36 | Male | 0.7 | Right lobe | I | Positive | Negative |
| 60 | 40 | Female | 0.3 | Left lobe | I | Negative | Negative |
| 61 | 30 | Female | 1.6 | Left lobe | I | Positive | Positive |
| 62 | 39 | Male | 1 | Right lobe | I | Negative | Positive |
| 63 | 40 | Female | 0.6 | Left lobe | I | Negative | Negative |
| 64 | 55 | Female | 0.3 | Left lobe | I | Negative | Negative |
| 65 | 44 | Female | 1 | Right lobe | I | Negative | Positive |
| 66 | 48 | Female | 0.5 | Right lobe | I | Negative | Negative |
| 67 | 36 | Female | 0.5 | Right lobe | I | Negative | Negative |
| 68 | 54 | Female | 0.2 | Left lobe | I | Negative | Negative |
| 69 | 42 | Male | 1.2 | Right lobe | I | Positive | Negative |
| 70 | 51 | Female | 0.2 | Left lobe | I | Negative | Negative |
| 71 | 56 | Female | 0.8 | Right lobe | I | Negative | Negative |
| 72 | 39 | Female | 0.2 | Right lobe | I | Positive | Negative |
| 73 | 34 | Female | 1.2 | Left lobe | I | Positive | Negative |
| 74 | 46 | Female | 2.5 | Left lobe | I | Positive | Positive |
| 75 | 43 | Female | 0.8 | Left lobe | I | Negative | Positive |
| 76 | 41 | Female | 1.5 | Right lobe | I | Positive | Positive |
| 77 | 40 | Female | 0.5 | Left lobe | I | Negative | Positive |
| 78 | 29 | Male | 0.5 | Left lobe | I | Negative | Negative |
| 79 | 28 | Female | 1.6 | Right lobe | I | Positive | Negative |
| 80 | 51 | Male | 1.5 | Right lobe | I | Negative | Positive |
| 81 | 51 | Male | 1 | Left lobe | I | Positive | Positive |
| 82 | 50 | Female | 0.4 | Left lobe | I | Positive | Negative |
| 83 | 30 | Female | 0.8 | Right lobe | I | Positive | Negative |
| 84 | 58 | Male | 0.4 | Left lobe | I | Negative | Negative |
| 85 | 31 | Female | 0.5 | Right lobe | I | Positive | Negative |
| 86 | 55 | Female | 0.6 | Right lobe | I | Negative | Negative |
| 87 | 24 | Female | 1.3 | Right lobe | I | Positive | Positive |
| 88 | 29 | Female | 1.5 | Left lobe | I | Negative | Negative |
| 89 | 47 | Female | 0.4 | Left lobe | I | Negative | Negative |
| 90 | 70 | Female | 0.8 | Left lobe | II | Positive | Negative |
| 91 | 37 | Female | 1 | Left lobe | I | Negative | Negative |
| 92 | 45 | Female | 0.6 | Left lobe | I | Negative | Negative |
| 93 | 32 | Female | 0.2 | Right lobe | I | Negative | Positive |
| 94 | 38 | Female | 0.5 | Left lobe | I | Negative | Negative |
| 95 | 38 | Female | 0.5 | Right lobe | I | Positive | Negative |
| 96 | 55 | Female | 0.5 | Left lobe | II | Positive | Negative |
| 97 | 49 | Female | 0.6 | Right lobe | I | Negative | Negative |
| 98 | 46 | Male | 3 | Right lobe | I | Positive | Positive |
| 99 | 33 | Male | 1.5 | Right lobe | I | Positive | Positive |
| 100 | 48 | Male | 1.5 | Left lobe | I | Negative | Negative |
| 101 | 53 | Male | 0.7 | Right lobe | I | Positive | Positive |
| 102 | 38 | Female | 1.2 | Left lobe | I | Negative | Negative |
| 103 | 26 | Male | 0.2 | Right lobe | I | Negative | Negative |
| 104 | 27 | Female | 0.6 | Left lobe | I | Positive | Negative |
| 105 | 50 | Female | 1 | Left lobe | I | Negative | Positive |
| 106 | 43 | Female | 1.3 | Left lobe | I | Negative | Negative |
| 107 | 43 | Female | 0.5 | Left lobe | I | Positive | Positive |
| 108 | 31 | Male | 2 | Left lobe | I | Positive | Negative |
| 109 | 50 | Male | 0.6 | Right lobe | I | Negative | Positive |
| 110 | 37 | Female | 1 | Left lobe | I | Negative | Negative |
| 111 | 34 | Male | 0.4 | Right lobe | I | Positive | Positive |
| 112 | 30 | Female | 0.5 | Left lobe | I | Negative | Negative |
| 113 | 32 | Female | 0.7 | Right lobe | I | Positive | Positive |
| 114 | 43 | Female | 1.2 | Right lobe | I | Positive | Positive |
| 115 | 43 | Female | 1.3 | Left lobe | I | Negative | Negative |
| 116 | 49 | Female | 1.5 | Left lobe | I | Positive | Negative |
| 117 | 45 | Female | 0.5 | Left lobe | I | Negative | Negative |
| 118 | 55 | Female | 0.6 | Right lobe | I | Negative | Negative |
| 119 | 43 | Female | 0.5 | Left lobe | I | Positive | Negative |
| 120 | 79 | Female | 1 | Right lobe | I | Negative | Negative |
| 121 | 33 | Female | 1.1 | Right lobe | I | Negative | Negative |
| 122 | 27 | Female | 0.5 | Right lobe | I | Negative | Negative |
| 123 | 52 | Male | 0.5 | Left lobe | I | Negative | Positive |
| 124 | 46 | Male | 0.5 | Right lobe | I | Negative | Negative |
| 125 | 41 | Male | 0.5 | Left lobe | I | Negative | Positive |
| 126 | 38 | Female | 0.4 | Left lobe | I | Negative | Negative |
| 127 | 50 | Female | 0.8 | Left lobe | I | Negative | Negative |
| 128 | 47 | Female | 0.6 | Right lobe | I | Negative | Negative |
| 129 | 35 | Female | 0.5 | Left lobe | I | Positive | Negative |
| 130 | 60 | Female | 2 | Left lobe | I | Negative | Positive |
| 131 | 34 | Female | 0.5 | Left lobe | I | Negative | Positive |
| 132 | 38 | Female | 0.8 | Right lobe | I | Negative | Positive |
| 133 | 64 | Female | 0.6 | Left lobe | I | Negative | Positive |
| 134 | 39 | Male | 0.8 | Left lobe | I | Negative | Negative |
| 135 | 30 | Female | 1.2 | Left lobe | I | Positive | Negative |
