# Supplementary material for: Intratumor Epigenetic Heterogeneity—A Panel Gene Methylation Study in Thyroid Cancer
Source: Front Genet. 2021 Sep 3;12:714071. doi: 10.3389/fgene.2021.714071 (PMC8446600; doi:10.3389/fgene.2021.714071)
Supplement: Supplementary Table 2 — Primers used for methylation analysis. [file Table_2.docx]

Supplementary table 2. Primers used for methylation analysis

| **Gene** | **Primers** | **Sense primer (5’-3’)** | **Antisense primer (5’-3’)** |  |
| --- | --- | --- | --- | --- |
| *MGMT* | U | TTTGTGTTTTGATGTTTGTAGGTTTTTGT | AACTCCACACTCTTCCAAAAACAAAACA |  |
| *MGMT* | M | TTTCGACGTTCGTAGGTTTTCGC | GCACTCTTCCGAAAACGAAACG |  |
| *TIMP3* | U | TTTTGTTTTGTTATTTTTTGTTTTTGGTTTT | CCCCCAAAAACCCCACCTCA |  |
| *TIMP3* | M | CGTTTCGTTATTTTTTGTTTTCGGTTTC | CCGAAAACCCCGCCTCG |  |
| *DAPK* | U | GGAGGATAGTTGGATTGAGTTAATGTT | CAAATCCCTCCCAAACACCAA |  |
| *DAPK* | M | GGATAGTCGGATCGAGTTAACGTC | CCCTCCCAAACGCCGA |  |
| *MLH1* | U | TTTTGATGTAGATGTTTTATTAGGGTTGT | ACCACCTCATCATAACTACCCACA |  |
| *MLH1* | M | ACGTAGACGTTTTATTAGGGTCGC | CCTCATCGTAACTACCCGCG |  |
| *TMEM176A* | U | GGAAGAAAGATGTTTTGTGGATAGGAT | CAACTAATATCCACTCTACTCAACCACA |  |
| *TMEM176A* | M | GAAGAAAGACGTTTTGTGGATAGGAC | CTAATATCCGCTCTACTCGACCGCG |  |
| *DIRAS1* | U | GTTTTTATTTTGAGGGAGGGT | ACCCCACACAAAACCCCTACC |  |
| *DIRAS1* | M | GTTTTTATTTCGAGGGAGGGC | GCCCCACGCGAAACCCCTACC |  |
| *sFRP1* | U | GTTTTGTAGTTTTTGGAGTTAGTGTTGTGT | CTCAACCTACAATCAAAAACAACACAAACA |  |
| *sFRP1* | M | TGTAGTTTTCGGAGTTAGTGTCGCGC | CCTACGATCGAAAACGACGCGAACG |  |
| *sFRP2* | U | TTTTGGGTTGGAGTTTTTTGGAGTTGTGT | AACCCACTCTCTTCACTAAATACAACTCA |  |
| *sFRP2* | M | GGGTCGGAGTTTTTCGGAGTTGCGC | CCGCTCTCTTCGCTAAATACGACTCG |  |
| *HIN1* | U | GAAGTTTTGTGGTTTTGTTTGGGTAGTT | CACACAAAACCCCAAAAAAACAACA |  |
| *HIN1* | M | GTTTCGTGGTTTTGTTCGGGTAGTC | GCAAAACCCCAAAAAAACGACG |  |
| *AP2* | U | GTAGTTTTATTTGGGTGTGAGATTGAG | ACACAAATAATCAAACCAACATCACA |  |
| *AP2* | M | GTTTTATTTGGGTGCGAGATCG | AATAATCGAACCGACGTCGCG |  |
| *ER* | U | TGTTGTTTATGAGTTTAATGTTGTGGTT | AAAAAAACCCCCCAAACCATT |  |
| *ER* | M | ACGAGTTTAACGTCGCGGTC | ACCCCCCAAACCGTTAAAAC |  |
| *DACT2* | U | TTGGGGTGTGTGTAGATTTTGTTTTTTGT | CCCAAACCCCACAAACAACACCA |  |
| *DACT2* | M | GCGCGTGTAGATTTCGTTTTTCGC | AACCCCACGAACGACGCCG |  |
| *CDH1* | U | GGGTTTTTGGAGTTGTAGTTTTTTGGT | CTCCCATCACTAAAAAATCCAAAACACA |  |
| *CDH1* | M | TTTGGAGTCGTAGTTTTTCGGC | CATCACTAAAAAATCCGAAACGCG |  |
| *RASSF1A* | U | GGGGTTTGTTTTGTGGTTTTGTTT | AACATAACCCAATTAAACCCATACTTCA |  |
| *RASSF1A* | M | GGGTTCGTTTTGTGGTTTCGTTC | TAACCCGATTAAACCCGTACTTCG |  |
| *SOX17* | U | TTAGGGGTGTTTGTAGTGTTATTAGGTT | TAAAACACTAAAATACCCCAAAAACTACA |  |
| *SOX17* | M | GGGGCGTTCGTAGTGTTATTAGGTC | AAACACTAAAATACCCCGAAAACTACG |  |
| *GATA4* | U | TTTGTATAGTTTTGTAGTTTGTGTTTAGT | CCCAACTCACAACTCAAATCCCCA |  |
| *GATA4* | M | GTATAGTTTCGTAGTTTGCGTTTAGC | AACTCGCGACTCGAATCCCCG |  |
| *BCL6B* | U | TTTTGTTTTGGATTTGTTATTTGGAGAGT | CTTAACCTCAACTCCTTTATCTAACCA |  |
| *BCL6B* | M | CGTTTTGGATTCGTTATTTGGAGAGC | TAACCTCGACTCCTTTATCTAACCG |  |
| *GPX3* | U | GTTTATGTTATTGTTGTTTTGGGAT | CACCATCCATCTAAAATATCCAACA |  |
| *GPX3* | M | TATGTTATTGTCGTTTCGGGAC | GTCCGTCTAAAATATCCGACG |  |
| *CRBP-1* | U | GTGTTGGGAATTTAGTTGTTGTTGTTTT | ACTACCAAAACAACAACTACCAATACTACACA |  |
| *CRBP-1* | M | TTGGGAATTTAGTTGTCGTCGTTTC | AAACAACGACTACCGATACTACGCG |  |
| *p16* | U | TTATTAGAGGGTGGGGTGGATTGT | CAACCCCAAACCACAACCATAA |  |
| *p16* | M | TTATTAGAGGGTGGGGCGGATCGC | GACCCCGAACCGCGACCGTAA |  |
| *RUNX3* | U | GTTGGTGGATTATGTAGGTGAGTTT | ACTCACCTTAAAAACAACAAACAACA |  |
| *RUNX3* | M | GCGGATTACGTAGGCGAGTTC | ACCTTAAAAACGACGAACAACG |  |
| *RARbeta* | U | TTGGGATGTTGAGAATGTGAGTGATTT | CTTACTCAACCAATCCAACCAAAACAA |  |
| *RARbeta* | M | TGTCGAGAACGCGAGCGATTC | CGACCAATCCAACCGAAACGA |  |
| *CDX2* | U | TTTTTTGTGTTTTTTGGTAGTTTTTAGT | TAACTCACATACATAATAACAAAAATCCA |  |
| *CDX2* | M | TTTTCGTGTTTTTCGGTAGTTTTTAGC | ACTCACGTACATAATAACGAAAATCCG |  |
| *WIF1* | U | GGTTTTTGAGTGTTTTTTTTTGGGTTT | AATACAATACACCCAATAAAACACCCA |  |
| *WIF1* | M | GTTTTTGAGTGTTTTTTTTCGGGTTC | AATACGATACGCCCAATAAAACG |  |
